# Glycoconjugate Nanoparticle-Based Systems in Cancer Immunotherapy: Novel Designs and Recent Updates

**DOI:** 10.3389/fimmu.2022.852147

**Published:** 2022-03-30

**Authors:** Joseph J. Barchi

**Affiliations:** Chemical Biology Laboratory, Center for Cancer Research, National Cancer Institute at Frederick, Frederick, MD, United States

**Keywords:** tumor-associated carbohydrate antigens, nanoparticles, immunotherapy, polysaccharide, polymers, size, tumor microenvironment, vaccine

## Abstract

For many years, cell-surface glycans (in particular, Tumor-Associated Carbohydrate Antigens, TACAs) have been the target of both passive and active anticancer immunotherapeutic design. Recent advances in immunotherapy as a treatment for a variety of malignancies has revolutionized anti-tumor treatment regimens. Checkpoint inhibitors, Chimeric Antigen Receptor T-cells, Oncolytic virus therapy, monoclonal antibodies and vaccines have been developed and many approvals have led to remarkable outcomes in a subset of patients. However, many of these therapies are very selective for specific patient populations and hence the search for improved therapeutics and refinement of techniques for delivery are ongoing and fervent research areas. Most of these agents are directed at protein/peptide epitopes, but glycans–based targets are gaining in popularity, and a handful of approved immunotherapies owe their activity to oligosaccharide targets. In addition, nanotechnology and nanoparticle-derived systems can help improve the delivery of these agents to specific organs and cell types based on tumor-selective approaches. This review will first outline some of the historical beginnings of this research area and subsequently concentrate on the last 5 years of work. Based on the progress in therapeutic design, predictions can be made as to what the future holds for increasing the percentage of positive patient outcomes for optimized systems.

## 1 Introduction

During the 21st century, major changes in the way we detect, treat and prevent disease have been developed and put into practice. This has been highly evident when it comes to cancer therapy: Novel and innovative directions in discovery have resulted in modalities to treat various malignancies in more selective and “personalized” ways (1). Three new advances that have led the way when examining the past 20 years of discovery come immediately to mind: 1) The ready availability of cancer genome sequences after unraveling the human genome at the turn of the century (2), 2) The advent of targeted therapy for cancer (3) and 3) The development of biologics, primarily in the various forms of immunotherapies (4) to fight disease. All of these advances, to varying degrees, embody what we now refer to as “personalized” medicine, where the ability to look deeply into the biology of individual tumors helps to define disease-relevant genetic mutations, biomarker expression or immunological signatures and tailor a particular therapeutic regimen to those modifications for optimized outcomes. In the past several years, researchers and clinicians alike have looked upon this concept as the “future” of medicine and medicinal chemistry, where the use of broadly non-selective acting cytotoxins and radiation treatments can be replaced with individualized (less cytotoxic and more effective) care. For many cases, this has been realized and lives have been saved or prolonged with high quality when this mode of treatment is successful. Some of the more notable success stories since 2001 are:

1) Starting in 2005, the development of The Cancer Genome Atlas (TCGA) has produced a compilation of cancer genomes from hundreds of different tumors for individualized biomarker discovery. These “Omic” studies have defined relevant cancer-specific genes that can be targeted by specialized therapy.
2) The development of the first anticancer *targeted therapy* to the *Bcr/Abl* gene product, a fusion protein that produces a constitutively active non-receptor tyrosine kinase that drives proliferation of Chronic Myelogenous Leukemia (CML) cells. Gleevec (Glivec), now called imatinb, was considered the first targeted therapeutic drug likened to what Paul Ehrlich referred to as a specific “bullet” that kills these (as well as some other) tumors (3, 5). Many derivatives of imatinib that target other driver kinases have been developed since that time, as well as a variety of small molecules targeted to other biomarkers determined from the work outlined in point #1.
3) The discovery that the human immune system can be harnessed to eradicate certain cancers, primarily through the inhibition of tumor-associated immunosuppressive mechanisms. So-called checkpoint inhibitors have been developed and FDA approved. These studies have paved the way for a host of monoclonal antibody therapies against these checkpoints, followed by the development of engineered T-cells from individual patient sera where a tumor-specific biomarker-binding molecule can direct these T-cells to a malignancy and exert a “dialed-in” cytotoxic effect on tumor cells (Autologous T-Cell therapy and Chimeric Antigen Receptor-T cells, or CAR-T cells) (6–8)

These three concepts have led to a series of newly approved drugs that have kept cancer at bay for thousands of patients whose disease course may otherwise would have been fatal. References to some success stories for cancer genomic studies can be found at the webpages for TCGA (9–14) at the Human Genome Research Institute of the NIH (https://www.genome.gov/Funded-Programs-Projects/Cancer-Genome-Atlas) and the National Cancer Institute webpage https://www.genome.gov/Funded-Programs-Projects/Cancer-Genome-Atlas). Targeted therapy has also seen a boon in approved agents, mostly derived from inhibition of driver tyrosine kinases that are overexpressed in various tumors (3, 15, 16). Couple the successful identification of tumor biomarkers by the TCGA with high throughput screening of drug candidates and optimization for specific protein binding sites and many additional tumor-targeted therapies will be available in the years to come. Cancer immunotherapy research has perhaps seen the steepest growth in the past decade. Approvals for new antibody and CAR-T therapies continue a remarkable pace, and refinement of molecular parameter such as the type of binding and regulatory proteins used to construct the T-Cell itself has helped to reduce off target effects and lower the threshold of activation necessary for optimal therapeutic efficacy.

However, as with all successes come setbacks and unanticipated factors that can continually be improved. For immunotherapy, these can be due to a host of factors, many that stem from a post-treatment overdriven immune system (cytokine release syndrome, activation of co-inhibitory pathways leading to T-cell exhaustion and effector cell neurotoxicity caused by overactive cytokine release in the cerebral spinal fluid) (17–19). Often, the immunotherapy is not enough to overcome the tumor immunosuppressive environment, led by tumor-associated macrophages and other lymphocytes that secrete cytokines that trigger the production of checkpoint molecules like PD-1 and PD-L1 (20–23). These drawbacks, however, have not dampened the enthusiasm for continued research to determine ways of more effectively utilizing the immune system to ward off tumor growth.

While most of the tumor-associated biomarkers, or “antigens” for CAR-T cell therapy are protein-derived, there is a separate family of molecules that are uniquely different on the surface of tumor cells compared to normal cells. These are the oligosaccharide structures that make up the various surface glycans that are present on every mammalian cell. Cell-surface glycans have distinct structural compositions on normal cells that dramatically change on transformed malignant cells. The aberrant structures are important partners in protein and cell binding events that lead to more aggressive tumor phenotypes. In addition, they are recognized by the immune system as “non-self”-like presentations and hence have been the basis of many tumor vaccine strategies. Tumor-associated carbohydrate antigens (TACAs) have been targets of therapeutic design in the fields of both anti-adhesive and immunotherapy for decades. Until relatively recently, TACAs had taken a back seat to protein antigens in cancer immunotherapeutic design, but there are now numerous reports on TACA-based drug and vaccine design as options in developing active or passive immune therapies to combat cancer.

Combined with targeting tumor-bearing carbohydrate structures, a valid strategy to both develop new entities for more selective delivery and overcome some of the other drawbacks of immunotherapy is to use platforms derived from various nanostructures. Nanotechnology in biomedicine has also seen and incredible boon during the last decades of personalized medicine. There is a plethora of novel platforms that have been developed both for delivery, targeting, self-degradation and tissue-selective targeting. Due to the heated scientific interest in the areas, there are also many recent reviews that have been published on these subjects, both separate and together (24–35). Thus, this review will first introduce the field and subsequently concentrate on the advances gained in the past 5 years. In a final discussion, an attempt will be made to critically assess the various platforms new available and offer opinions as to which are the most promising for future development.

## 2 A Brief History of Cancer Immunotherapy

Many treatises about the history of cancer therapy have been written and trace back the beginnings of cancer immunotherapy thousands of years (36). This is quite remarkable as these accounts detail spontaneous tumor regressions after some type of microbial (viral or bacterial) infection. The accompanying high fever and undoubtedly inflammation caused by the infection led to a strong immune response and subsequent remission of the invading neoplasm. This was the basis for several experiments performed by William Coley in the late 19th century, where he capitalized on case studies of patients who were cured of their tumors when infected with a streptococcal bacterium that causes the skin infection erysipelas (36). Inoculation of cancer patients with a combination of two separate attenuated bacterial strains caused remarkable remission rates compared to many of the medical treatments available at that time. Thus, “Coley’s Toxin”, which was a combination of both gram-negative and gram-positive bacteria could arguably be considered the first “adjuvanted” vaccine to treat tumors. Many of the immune-stimulatory molecules that are used today in vaccine adjuvants come from the cell walls of these bacteria, which serve to kick-start the immune system, most likely leading to the activation of stimulatory signals and the production of various immune cells, modulators and cytokines. Unfortunately, the use of bacterial toxins as (immuno-)’therapy” was quickly frowned upon, especially since the mechanism of tumor regression was not well understood at the time. Other modalities, such as radiation and newly developed small molecule drugs remained the fashion until almost 100 years later and the discovery of interferon and other cytokine immune stimulants like Interleukin-2 (IL-2) (37–41). While IFN and IL-2 were initially touted as game changers in tumor therapy, they came crashing to unceremonious endings based on unexpected toxicities and paradoxical immune-suppression mechanisms in the clinic. The end of the 20^th^ century saw several other milestones in cancer immunotherapy, such as the discovery of first dendritic cells (42) and then natural killer cells (43); the theory of tumor immunosurveillance; the use of the Bacille Calmette-Guérin (BCG) tuberculosis vaccine to be used against tumors in mice (44), and the first cancer vaccine composed of an adjuvanted tumor lysate as early as 1959.

**Figure 1:**
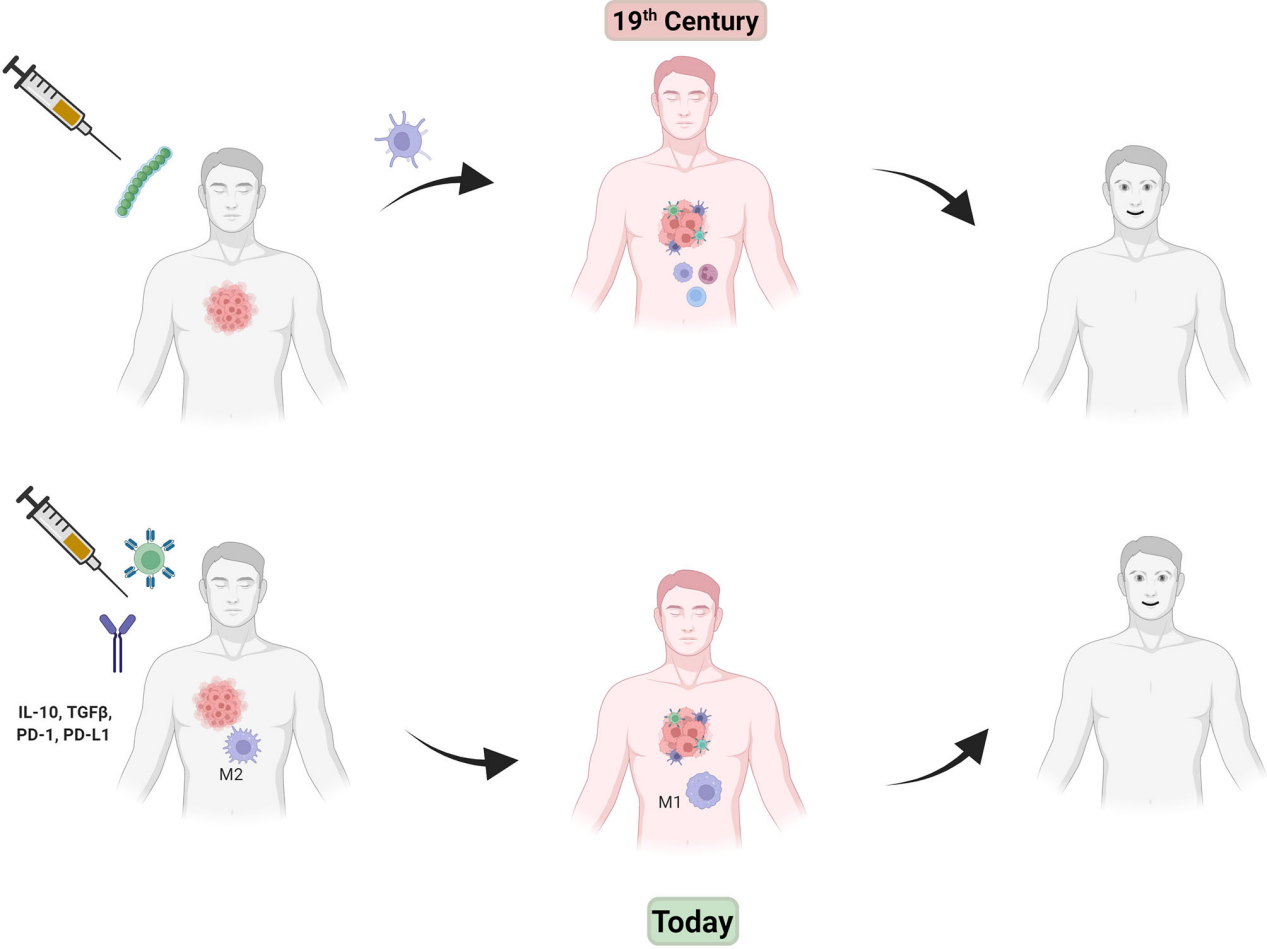
In the late 19^th^ century, injection of Coley’s toxin (green beads, top left) caused an inflammatory immune response (center drawing, red man with immune cells attacking tumor) that helped tumor regression. Modern day immunotherapy (lower drawings, left to right) shows injection of CAR-T cells (green spiked ball) and/or checkpoint inhibitor antibodies (IgG figure) results in a similar effect without the use of toxic bacteria.

While this smattering of advances could be thought of as highly promising, little follow up and hence a lack of actual developed products left most clinicians disinterested. Fast forward to the 21^st^ century and a revolution has now taken hold with a multitude of studies anointing cancer immunotherapy as the “fourth horsemen” of antitumor therapy along with (now more highly precise) surgery, chemotherapy and radiation (45). By the 2000’s it was well known that the immune system can have a powerful effect on tumor growth and dissemination. But perhaps the two discoveries, or re-discoveries as it may be considered, that brought the concept back to the forefront of cancer biology were: 1) unraveling the details of the immunosuppressive tumor microenvironment and that countering that immunosuppression is a viable therapeutic strategy and, 2) that a patient’s own T-cell population could be tailored to fight their tumor by reengineering these cells ex-vivo followed by reintroduction into the patient. **Figure 1** shows a schematic of “then and now” in cancer immunotherapy.

Point #1 magnifies the difficulties faced by a patient’s immune system in eradicating a tumor entirely. Tumors are not isolated islands unto themselves but are bathed in an environment that contains components that can be beneficial to tumor growth and hostile to any antitumor immune response (the tumor microenvironment). Various phenotypes such as Regulatory T-cells, M2 macrophages and mononuclear-derived suppressor cells (MDSCs) send out negative signaling to suppress the immune response and lead to the “exhaustion” of the T-cell response that would otherwise be antitumor (46). In normal tissue, these serve to halt an immune response that may proceed unchecked, essentially “putting the brakes” on a system which when dysregulated could cause detrimental pathologies such as autoimmune disease. In the tumor microenvironment, engagement and signaling through these checkpoints serve to allow proliferation and tumor invasion to proceed; hence the idea that inhibition of these would “remove the brakes” on the antitumor immune response. This has been realized with approved antibody therapies against regulatory proteins such as CTLA-4 (Ipilimumab) (47), PD-1 (Nivolumab) (48) or its ligand PD-L1 (Atezolizumab) (49). These approvals ignited several novel studies into either inhibition of the tumor suppressive environment or enhancing a natural antitumor immune response. Subsequent work on Point #2 above led to the discovery that, engineering a patient’s own T-cells with a binding molecule (antibody fragments such as an ScFv) to a tumor-specific antigen (a so-called neoantigen) and linking this to signaling domains that promote T-cell proliferation after neoantigen engagement, could eliminate certain tumors. The ScFv portion denoted as a Chimeric Antigen Receptor (CAR) spawned an entire new field of study and the development of many new therapies called CAR-T cells (7, 50). These can be specifically tailored to mutated or overexpressed antigens on a tumor and have, to date, been highly successful against particular subsets of tumors, especially lymphomas. CAR-T’s against the CD-19 protein have been approved for acute lymphoblastic leukemia (ALL, Kymriah) (51, 52) or other types of B-cell lymphomas (Yescarta) (53). Along with all the benefits of these therapies, they also come with (perhaps not unexpected) drawbacks. Ever since the amazing results obtained by Coley’s Toxin, adverse effects (AEs) have been a serious issue with immunotherapy. By turning off regulatory mechanisms, the immune system can go into overdrive causing issues such as Cytokine Release Syndrome (CRS)—an inflammatory process that can cause high fever and sometimes organ failure (18, 50, 54). This is part of what is sometimes referred to as a “cytokine storm” reaction to a pathogen or in the present discussion, CAR-T or checkpoint inhibition therapy, where a marked dysregulation of cytokine production can lead to fatal outcomes (18). This has been seen in the recent severe cases of SARS-CoV-2. An informative review of cytokine storm and CRS was recently reported by Fajgenbaum and June (55).

Even with the possibility of severe adverse effects, CAR-T and anti-checkpoint therapy have become standard for a select group of tumor types (primarily hematological), but researchers and clinicians alike are working feverishly to design these agents with lower risk of AE’s and increase their effectiveness in solid tumor immunotherapy. With regards to cancer vaccines however, while many designs have shown a degree of clinical effectiveness, the success of specific platforms has been much more difficult to realize (56, 57). To date, there are only a small handful of cancer vaccines that are used clinically, and these are split between therapeutic and preventative vaccines. Preventative vaccines are active against specific microbes (bacteria or viruses) that are the cause of or have a role in the development of a specific malignancy. The BCG vaccine described above that was originally developed against tuberculosis, is used in the treatment of bladder cancer (58–63). HEPLISAV-B is a vaccine against hepatitis B virus, whose infection leads to many hepatocellular carcinomas (liver tumors) (64, 65). Gardasil and Cervarix are vaccines against the Human Papilloma Virus (HPV) which is known as the causative agent in cervical cancer (66–68). Gardasil comes in two different polyvalent formulations which are each active against several different serotypes of HPV (69–71). Cervarix is active against the most common forms type 16 and 18 (71, 72). Sipuleucel-T (Provenge) is a therapeutic vaccine that is approved to treat metastatic castration-resistant prostate cancer (73). It is the only approved dendritic cell vaccine that uses the patient’s own cells, re-engineered to overexpress prostatic acid phosphatase (PAP), an antigen on prostate cancer cells, and an immune stimulating cytokine, Granulocyte-Macrophage Colony Stimulating Factor (GM-CSF). Although FDA approved as a therapy, Provenge increases survival of prostate cancer patients by only 4 months. The only other therapeutic vaccine, Talimogene laherparepvec (T-VEC) is a hemolytic virus formulation that infects tumors and causes subsequent lysis. A herpes simplex virus was reengineered where two genes were deleted (Infected Cell Proteins (ICP) 34.5 and 47) and one for GM-CSF was added (74, 75). This allows the attenuated virus to infect tumors and still remain replication competent; after replication in tumors, those cells burst, thereby killing the cell and in the process releasing tumor antigens that can be processed and presented to T-cells. T-VEC is the only oncolytic virus therapy formally approved for cancer treatment, but only in specific instances of inoperable melanoma. The vaccine is injected intratumorally, that is, directly into the tumor tissue itself, since these are uniquely difficult to treat malignancies.

It is obvious that the last 20 years has seen a great increase in advances in tumor immunotherapy, with some relatively astounding results. In addition, CAR-T cell and checkpoint therapy are being continually approved for different tumor types and on clinical trials are being designed at a rapid pace.

Work is continuing to refine how to best design CAR-T cells with lower AE’s, better selectivity and higher efficacy in solid tumors. Reprogramming of the immunosuppressive tumor microenvironment is also an area ripe for further investigation. Lastly, progress in appropriate vaccines where agents can advance past Phase III trials has so far been what could be considered unsuccessful (76, 77), and hence a much more focused research effort to advance this this form of active immunotherapy is desperately needed.

The remainder of this review will focus on how nanotechnology and novel nanoparticle platforms can inform the future success of vaccine and immunotherapeutics in cancer. This will be discussed in the context of using TACAs and other glycans as targets for immune stimulation, presentation and delivery systems. The preparation of glycoconjugates in nanoparticle design will also be discussed.

## 3 Glycoconjugates, Antitumor Therapy and Nanotechnology

Cellular glycans are ubiquitous in all living organisms and it follows that they would be involved in a variety of important functions. This invariably includes the immune response and its modulation: Certain signaling pathways are mediated through the expression and interactions of specific glycan structures on cells of the innate and adaptive immune systems. They serve to both initiate disease states such as autoimmune diseases, cancer and those caused by microbial infections. They can also subsequently contribute to the progression of these states, such as cancer metastasis and advanced stage rheumatoid arthritis. As mentioned above, TACA’s and tumor cell surface glycans on glycolipids, glycoproteins and proteoglycans are all aberrantly expressed relative to a normal cell phenotype. This imparts alternate cellular properties to tumors relative to normal cells such as different adhesive, migration, and signaling abilities. Thus, cellular glycans, and as it would follow, the proteins that interact with these structures (lectins, such as the Selectins, Galectins and Siglecs) have been targets of anticancer therapy for many years (78–81).

TACAs are considered antigens since the immune system mounts a response to them, even though they are “self” glycans. Carbohydrates are considered T-cell independent antigens since the standard immunological response to these structures alone is humoral with no stimulatory signals that can promote T-cell production and expansion. Foreign carbohydrate antigens on bacteria and viruses are recognized by immunoglobulin receptors on B-cells to initiate antibody production usually through non-class-switched, low-affinity IgM production. Based on this model, carbohydrate-based vaccines (in particular those to bacterial capsular and cell-surface glycans) have traditionally been designed as conjugates to carrier proteins whose primary sequences encode T-cell epitopes to help foster a cell-mediated response through presentation by Major Histocompatibility Complexes (MHC), primarily CD4^+^ helper T-cells. Most vaccines against various bacterial infections are constructed in this way, with proteins such as tetanus toxoid or the non-toxic mutant of diphtheria toxin, CRM197, used as T-cell epitope “carriers” (82, 83). Not surprisingly, this design strategy has been coopted for the construction of cancer vaccines to tumor glycan-based antigens (80, 82–97). However, glycopeptides derived from cell surface proteins containing TACAs (or other immunogenic glycans) can also be presented to T-cells and the sugar can either contribute or detract from MHC binding (85, 98–110). Discovered about 25 years ago, another set of MHC-II-like antigen presenting molecules, the CD1 family (CD1a-CD1e), has been shown to present cellular and foreign glycolipid antigens to either γδT-cells or Natural Killer T-cells (NKT cells). The marine derived α-galactosyl ceramide was initially identified as a ligand for CD1d, and this discovery has spawned an entire research direction to define the features of and discover new glycolipid molecules that can be used as adjuvants in vaccine design (83, 111–114). More recently, the discovery of zwitterionic polysaccharides (ZPSs) from certain bacteria as polymers that can be presented to T-cells and elicit a cell-mediated response has shifted the paradigm of saccharide immunity (100, 115–117). Data in the last decade has shown that processing of ZPSs is through pathways similar to protein presentation, with breakdown of the polysaccharide after endocytosis and processing by MHC-II to present to CD4+ T-cells. ZPSs are part of the innate immune response after the discovery that they interact with and agonize toll-like receptor-2 (TLR2) signaling (118–120). The immunogenicity of certain TACAs has been enhanced by conjugation to ZPSs (121–123).

**Figure 2:**
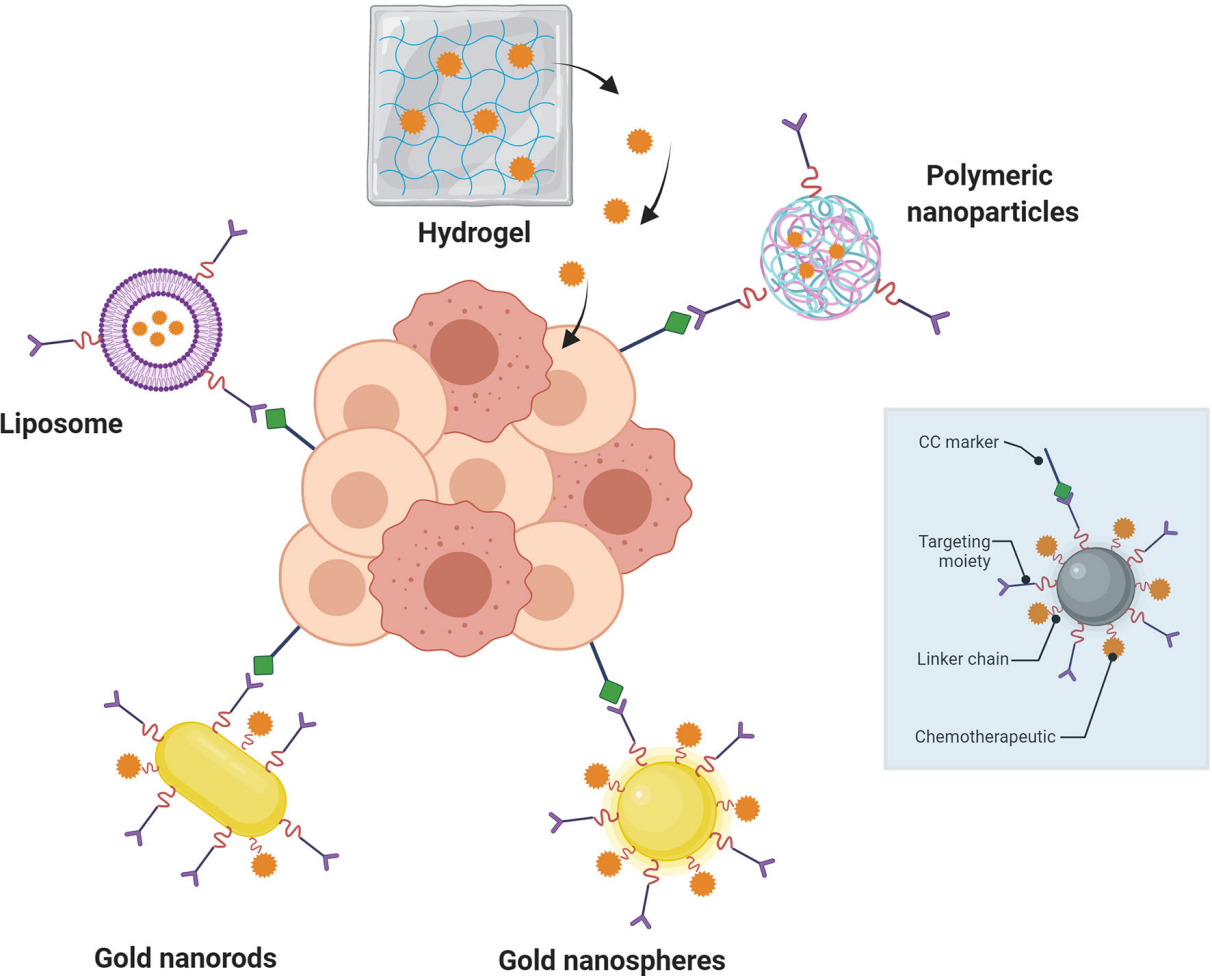
Nanoparticle-mediated targeted drug delivery to cancer cells (center, darker pink circles). Nanoparticles (blue box) containing targeting moieties bind to cancer cell (CC) markers on tumors to inhibit/agonize signaling or protein binding and/or deliver therapeutic drugs (red jagged spheres; e.g., small molecules or antibodies). Linkers are important components of the design for distance and physiochemical property requirements.

The citations in the previous paragraph clearly show that the use of TACAs and other tumor-associated glycans is a very active area of research and will continue to contribute to advances in immunotherapeutic against many tumor types. The problems of appropriate antigen design, tumor-selective biomarker selection and delivery systems for appropriate efficacy remain conceptually difficult to overcome. A direction that the field has followed for almost two decades now is to use nanotechnology (particles, polymers, colloids, carbon allotropes, etc., with individual core particle sizes in the 1-100 nm range) in its various forms to solve some of these issues. This can manifest itself in many ways, such as (**Figure 2**): 1) the use of different nanoparticles as (targeted) drug or antigen carriers, 2) taking advantage of nanoparticle size or shape to selectively home to tumors, 3) employ the multivalent presentation that nanoparticles can facilitate to enhance a particular response or 4) encapsulate therapeutic materials in a nanoshell for protection from degradation or metabolism before reaching an intended target tissue/cell. In the past few years, there have been may reviews written on the subjects contained in this manuscript, i.e., cancer immunotherapy and the use of nanotechnology in this quest. A search of the Web of Science using the terms “Cancer” “Immunotherapy” and “Review” reveals more than 17,000 articles, with >30 of them to be published in the year 2022 (the year after this review is being written()!… These references are relevant to this review (124–140)). The purpose of this review is thus to concentrate specifically on what may be the most useful technology developments in the last 5-7 years, focusing only on the use of nanoparticles in tumor immunotherapy approaches, as opposed to simple drug delivery and nano-platform development.

## 4 Glyco-Nanotechnology and Cancer Immunotherapy

As alluded to above, in 2021 there is no shortage of reviews published on the cancer immunotherapy, with or without the use of nanotechnological platforms. An attempt will be made here to outline the latest advances in the use of glycoconjugate nanoparticles and nanosized-polymeric systems in cancer immunotherapy while highlighting unique details of each with regards to innovation and future therapeutic potential.

**Figure 3:**
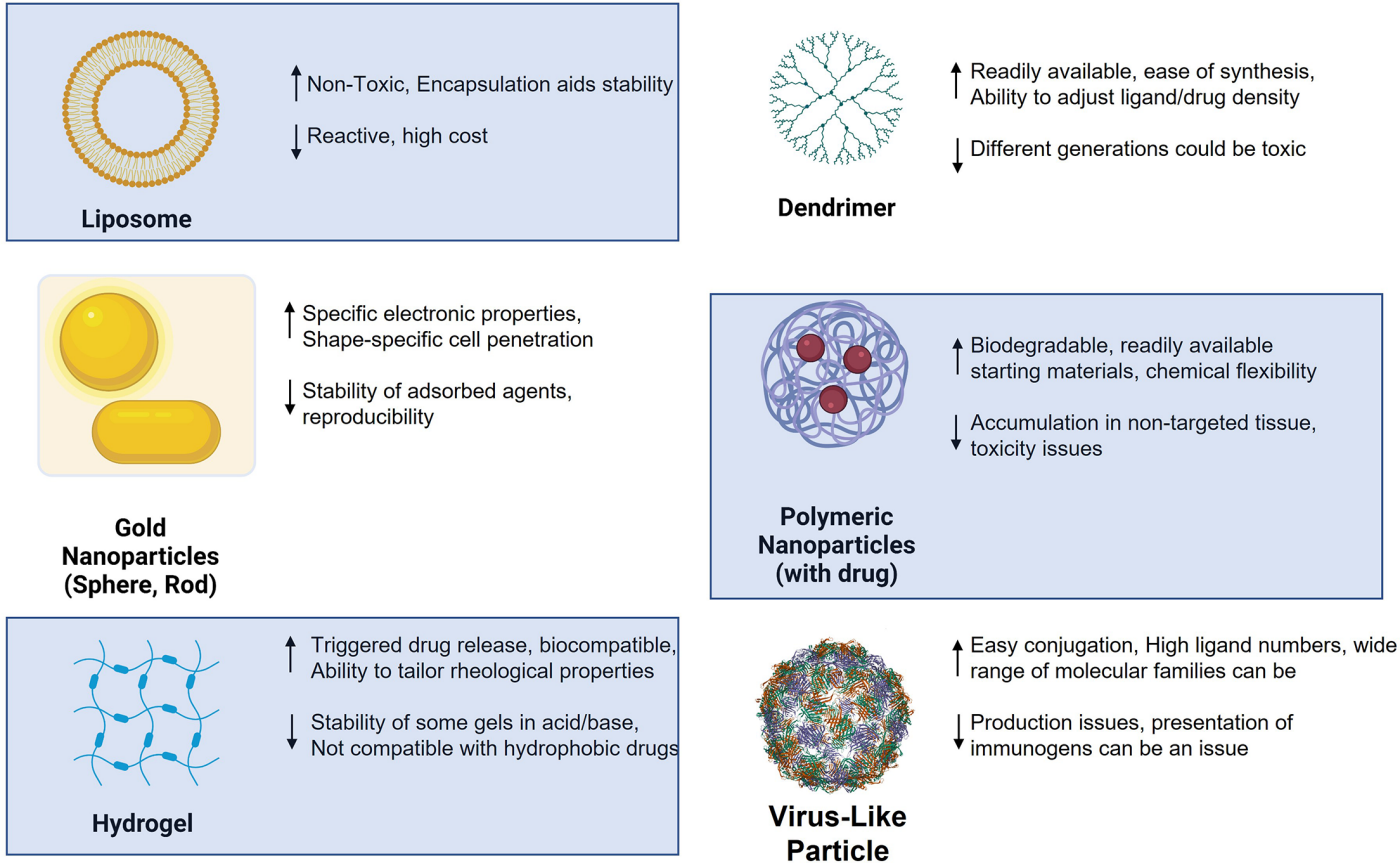
Various nanoparticles used in antitumor therapy design and selected advantages (up arrow) and disadvantages (down arrow).

Nanoparticles (NPs) have now been used in the design of new medicinal agents for more than two decades. There are both advantages and disadvantages to using nanoparticles over monomeric and single agent materials as a drug/vaccine, where it can be argued that their benefits outweigh their shortcomings. **Figure 3** illustrates some of the most widely used platforms in the past 20 years, highlighting some of their plusses and minuses for use in generic drug delivery or immunotherapy. Nanoparticles in immunotherapy have primarily been used in two related ways: 1) as drug delivery agents and 2) as vaccine platforms for the delivery of antigens and/or adjuvants to specific immune cells. The major advantages to using nanotechnology in therapy are the following: 1) NPs change both pharmacokinetic and pharmacodynamic properties of whatever entity they carry, leading to altered metabolism and often protection from degradation; 2) This added protection coupled with targeted delivery will improve the likelihood that the NPs go where they are supposed to and present antigens and adjuvants to specific immune cells and 3) the combination of (1) and (2) will reduce off- target effects. While there are many examples of the use of NPs in immune therapy, only a small percentage of those relate directly to glycoconjugates or the use of carbohydrates as antigens or ligands in the potential therapy.

As shown in **Figure 3**, certain families of NPs have distinct properties that are highly relevant to the interaction of glycans with their receptors. Paramount of these features is the inherent multivalent nature of NPs. NPs have a high surface area and multiple copies of carbohydrate ligands and/or antigens can be attached to different particle types. As alluded to in the figure, polymeric and dendrimeric particle surface density can be controlled and adjusted with high accuracy, as compared to, for example, inorganic nanoparticles where this ligand density could be more random and difficult to alter. It is a well- known property of carbohydrate-protein(lectin) binding that multivalency (the “Velcro” effect) is critical for high avidity binding and the elicitation of a subsequent biological effect (141). Thus, the use of nanosystems for immunization or ligand binding to lectins holds an inherent advantage to single molecule strategies.

### 4.1 Vaccines: Delivery Platforms

As alluded to earlier, many TACAs and glycolipid glycans have been the subject of anticancer vaccine development going back to the 1970’s starting with George Springer (142, 143). He discovered that the Thomsen nouveau (Tn, GalNAc-α-O-Serine/Threonine) and Thomsen Friedenreich (TF, Gal-β-1,3-GalNAc-α-O-Serine/Threonine) antigens were actually TACAs and that the body raised an immune response to them. A vaccine preparation of depleted red blood cells carrying the TF antigen that he developed was used by his 32 patients, where over half survived after 5 year and 7 survive over 10 years (144). Some patients had late-stage disease that at the time would not have lasted more than a few months. While cell preparations are in the micron size range and thus not “nano” (1-100 nm), this still could well be considered the beginning of particle-based glycoconjugate vaccine anticancer therapy. The following discussion will highlight some of the specific systems that have been successful in tumor immunotherapy.

#### 4.1.1 *Liposomes*

Nanosystem vaccine preparations to TACA’s and other glycans have a rich history dating back about 20 years. In 2005, the Boons group at the University of Georgia prepared a 3-component vaccine comprised of the Tn antigen, a T-cell epitope from poliovirus and a Toll-Like Receptor-2 (TLR2) agonist Pam3Cys lipopeptide linked covalently into one combined molecule (145). Incorporation into liposomes (constituting a “nano”vaccine) and immunization of mice revealed a robust humoral immune response. Refinement of this design strategy over the next several years showed that addition of a MUC1 peptide-conjugated Tn antigen dramatically increased the antibody titers (146–148); use of a 2,6-sialyl-Tn antigen also showed robust immune response to this antigen (149) and replacement of the poliovirus T-cell epitope with a longer MUC1-derived glycopeptide containing specific epitopes could also elicit a T-cell response similar to the original design (150). Other liposomal preparations have also been used in this context. One strategy showed that addition of galactosyl ceramide (*vide supra*) into the liposome could potentiate the immune response to a MUC1 glycopeptide (151). A recent interesting study by Shiga, et al., examined the effect of adding trehalose 6,6′-dimycolate (TDM) to a cationic liposomes on the antitumor immune response toward various cancers. TDM is a component of the cell wall of the bacterium BCG (also, *vide supra*) that is used as a treatment for bladder cancer (152). The idea was to use a subunit of that bacterium in place of the whole microbe as a non-toxic replacement that can be incorporated into various nano-formulations. This construct proved to be as or more active than BCG in mouse models of several cancers. This effect was reduced in knockout mice depleted in CD8+ cells and the C-type lectin Mincle, a receptor for TDM, suggesting interaction with Mincle was a prerequisite to biological activity. This simplified design may be useful in future vaccine constructs bearing tumor-associated antigens. An interesting study was reported by Yanigahara, et al., where a β1,3-β1,6-branched β-glucan called Aquaβ, chemically-derivatized containing pH sensitive glutaric acid esters were incorporated into liposomes (via conjugation of a percentage of the glutaric acid moieties with decyl amine groups) loaded with model antigen (OVA) (153). The strategy entailed interaction of these nanoparticles with C-type lectins or TLR proteins on the surface of APCs that bind β-glucans, such as Dectin-1 or TLR2, which leads to endocytosis and cross presentation of protein antigen to elicit a cell-mediated (CD8^+^) immune response. The response was greater with the branched polysaccharide relative to similarly-modified linear curdlan derivatives. The study suggests that β-glucans can be heavily derivatized and still maintain their APC activating properties.

The reader is referred to the following reviews for more information on the use of glycan-modified liposomes in immunotherapy (24, 154–156).

#### 4.1.2 Inorganic Nanoparticles

The use of metal-based 3-dimensional self-assembled monolayers and other inorganic particles has been a mainstay of tumor immunotherapy for several years. In particular, gold and iron oxide glyconanoparticles have been used as both diagnostic and therapeutic agents in a variety of antitumor research studies. The reader is referred to recent reviews concerning the use of glyconanotechnology in cancer therapy (24, 26, 28, 29, 31, 32, 34, 157).

##### 4.1.2.1 Gold Nanoparticles

Along with use as a novel drug delivery platform, gold nanoparticles alone can act as an immune stimulant and adjuvant for other immune-based therapies. Over the past 15 years, a series of papers were published by the Dykman group whose work established the immune functions of naked and neutral-passivated AuNPs (158–161). A review by this group in 2010 (160) seems to establish the concepts that, 1) colloidal gold could act as a carrier of various immunogens, including small molecules haptens to stimulate an antibody response, and 2) That colloidal gold nanoparticles can act as adjuvants on their own, but there is also contradictory reports on whether or not AuNPs themselves are actually harmful or offer some benefit to an organism through stimulation of immune cell proliferation.

Gold Glyco-NPs (AuGNPs) were developed over 20 years ago by the Penades group (162, 163), and we (164) and others (165) had contributed to the preparation of vaccine construct based on AuGNPs bearing either a single TACA or glycopeptides from cell surface mucins bearing TACAs. With a focus on work performed since ~2015, there have been some successful applications of this design as a tumor immunotherapy. An excellent review by Ferrando, et al., has recently been published (166) that outlines the use of AuGNPs as vaccines against bacterial and viral infections as well as cancer. Some of the most important heavily glycosylated tumor-associated antigens studied today are Mucins, and of those MUC1 is arguably the most heavily researched. The tandem repeat (TR) sequence of human mucins is a repeating segment of between 16-24 amino acids that extend out from the cell surface. These motifs are replete with serine and threonine residues that are mostly O-glycosylated (“Mucin-type” glycosylation) with different core structures. In tumors, these are truncated to 1-3 saccharide units comprising the aforementioned Tn, TF and sialyl-Tn/TF structures. These have been the subject of tumor vaccine studies for over 25 years. While the individual glycans were thought as the actual antigenic structures and originally used as conjugates to carrier molecules, it has become widely accepted that the context of the saccharide in the mucin environment—i.e., covalent attachment to the peptide backbone and presentation amidst other TRs—is the structure that more closely resembles the actual presentation on the cell surface. Thus, we and others pursued tumor/mucin-associated glycopeptides as the appropriate haptens for immunization.

**Figure 4:**
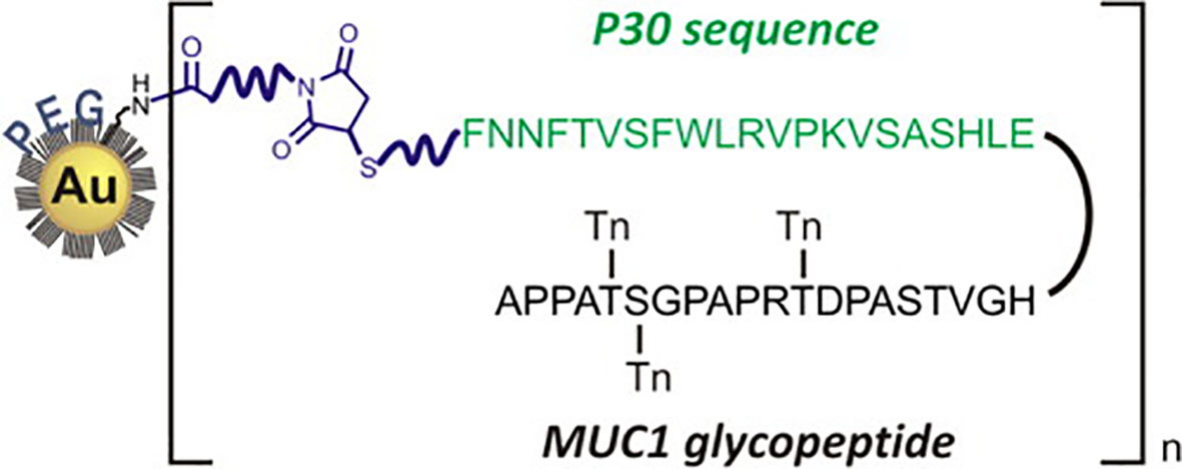
Design of Westerlind, et al., of PEGylated AuNPs with covalently-linked TACA glycopeptide coupled to the Tetanus Toxoid T-cell motif P30 (reprinted from reference 163).

The Kunz group has pioneered the synthesis and vaccine preparations of TR mucin glycopeptides from both MUC1 and MUC4 (104, 106, 167–170). Working with the Westerlind group, these researchers have reported on the immune evaluation of selected MUC1 glycopeptides coated on AuNPs (171). The unique design of their system, somewhat akin to that of Cameron, is shown in **Figure 4**.

Polyethylene Glycol (PEG) units functionalized with thiols at one terminus and amines or carboxylates at the other, were first used to passivate 13 nm AuNPs. A combined MUC1 glycopeptide/CD4+ helper sequence (P30 from Tetanus Toxoid) was synthesized by solid phase peptide synthesis with a C-terminal thiol. Addition of a NHS/maleimido heterobifunctional linker to the pegylated AuNP appended a maleimide unit that could be conjugated by the thiol-terminated P30-MUC1 glycopeptide. Immunization with complete Freund’s adjuvant and evaluation of the antisera showed IgG that react with the glycopeptide immunogen. Immunization with the combined peptide alone without the AuNP carrier was about 10-fold less potent. While the titers were albeit relatively low, and only three mice were used per immunization, the design may be useful for further development.

Our own recently published work showed that preparation of AuNPs with β-glucans (immune stimulating polysaccharides from fungal and cereal cell walls) and the MUC4 glycopeptide discovered in previous work elicited a strong immune response in animals with antibody titer up to 300,000 (172). A T-cell mediated response was also evident from a EliSpot assay where cytokine secretion showed evidence of glycopeptide presentation to T-cells through an MHC-II-dependent process.

##### 4.1.2.2 Iron Oxide Particles

Iron oxide NPs are easily constructed and can act as either carriers of various molecular families, similar to AuNPs, as well as act as contrast agents in Magnetic Resonance Imaging (MRI)-based diagnostic studies. They are often called Superparamagnetic Iron Oxide NPs (SPIONs) due to their specific properties such as nanometer size- and temperature -dependent magnetic fluctuations and the ability to be magnetized to high magnetic susceptibility from an external source. The Huang group pioneered the preparation and use of “glyco”-based SPIONs with a 2010 report that showed hyaluronic acid-coated SPIONs can maintain their CD44 binding properties while also being able to bind to and enter macrophages, allowing imaging of these cells. In 2015, this same group prepared lipopeptide-coated SPIONs also coated with TACAs for delivery to the immune system. In a unique design, they prepared SPIONS coated with functionalized oleic acid and MUC1 glycopeptides terminated by a phosphatidylethanolamine that “intercalated” into the hydrophobic SPIONs. These particles were able to activated dendritic cells in the presence of the TLR4 agonist monophospholipid A (MPLA). Immunization of mice elicited a strong IgG antibody response where the antisera could bind MUC1-presenting MCF-7 breast cancer cells and cause complement dependent cytotoxicity of these cells. This only occurred with the nanoparticles and not the glycolipopeptide alone. The authors speculated that the as-designed amphiphilic nature of the nanoparticles help them traffic to lymph nodes and hence trigger a better response. Nativi’s group synthesized standard ferromagnetic iron oxide nanoparticles as well as dextran-coated IONs displaying a mimetic of the Threonine-linked Tn antigen (173). These particles were able to trigger TNF-α gene expression in mouse macrophages with the same potency as LPS. Treatment of PBMCs with the dextran particles triggered the secretion of IL-6 and IL-10, similar to treatment with LPS. This activity only occurred when using the multivalent display on the nanoparticles and not with the monovalent mimetic.

#### 4.1.3 Virus-Like Particles

**Figure 5:**
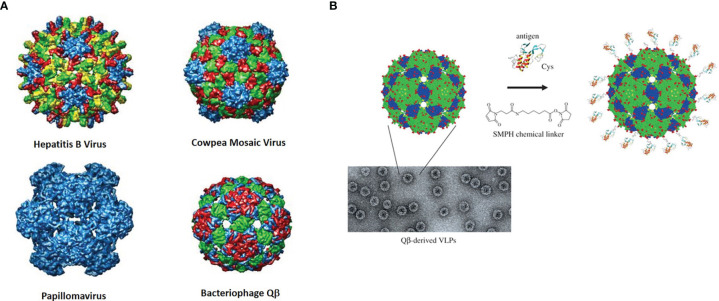
**(A)** Rendered crystal structures of different VLPs with color-coded rendered subunits. **(B)** Depiction and micrograph of Qβ and one example of how the surface may be modified with a bifunctional linker (with permission from “Therapeutic vaccines for chronic diseases: successes and technical challenges” **2011**, *366*, 2815-2822, DOI: 10.1098/rstb.2011.0103).

Virus-Like Particles (VLPs) are basically viruses that are devoid of genetic material and hence are replication incompetent. They can be isolated from naturally sources or prepared in different ways such as self-assembly through recombinant technologies. Capsid proteins and other can be used for their construction, but they can be produced in different organisms. Hence, VLPs come in a variety of “flavors” and are derived from a wide variety of virus families, including bacteriophages. The protein coats on their surfaces have several reactive functional groups exposed, such as amines and thiols, that can be conjugated with antigenic molecules (**Figure 5**).

The Huang group again has done some pioneering work in this area. They initially showed in 2013 that a simple monomeric Tn molecule displayed on the surface of bacteriophage Qβ can stimulate a powerful immune response to the GalNAc-glycoamino acid with antibody IgG titers close to 1,000,000 (174). the strength of the response was dependent on the dose of the vaccine as well as the density of antigen presented on the VLP. A glycan microarray analysis of the antisera showed that it was specific for Tn-containing structures.

A follow up to this work was published in 2019 that used the TF and Sialyl-Tn antigens as glycans coupled to the same MUC1 peptide sequence followed by attachment to Qβ (175). Again, incredibly high titers [>>10 (6)] were generated in ELISA assays to the constructs conjugated to BSA. The sera from the TF-conjugated glycopeptides recognized multiple MUC1 isoforms, weas cytotoxic to MUC1-bearing tumor cells and protected mice from tumor challenge post vaccination. Thus, the possibility of a preventative vaccine derive from these bacteriophage particles is a distinct future possibility. A final study with similar experimental details looked at the β-Threonine-linked TF glycopeptides as a possible solution to the inherent instability of the natural α-threonine-linked to endogenous glycosyl hydrolases (176). Similar antibody titers elicited for this construct in a MUC transgenic mouse model, and interestingly, these sera cross-reacted with the α-linked TF glycopeptides and recognized tumor cells with natural a-linked TF antigen. This “mimetic” design may prove very useful, as the β-linked molecules could be considered “foreign” in a tumor microenvironment setting. A study by Sartorius, et al., used variation on this theme by attaching an adjuvant – the aforementioned α-GalCer – to a VLP and examined the stimulation of invariant NKT (iNKT) cells (177). Bacteriophage loaded with α-GalCer stimulated activation of iNKT cells *in vitro* and *in vivo*. If these VLPs were programmed to display an immunogenic OVA peptide and then coated with α-GalCer they were found to stimulate CD8+ cell production and also protect mice form B16 melanoma. Given the success of bacteriophage particles in some of these studies, it is highly encouraging that the combination of adjuvant/antigen can be a successful strategy to construct a therapeutic vaccine. This may be useful with the MUC1 or other mucin-derived glycopeptides to avoid admixing of adjuvant and antigen. We used a similar strategy in our original AuNP vaccine design (164).

#### 4.1.4 Polymeric Nanoparticles

Since the two most abundant organic materials on earth are polymers of carbohydrates (cellulose and starch), it is no wonder that the study of the chemistry and potential biological applications of these types of molecules would encourage fervent research in this area. Glycopolymers in biomedicine also have a rich history, with a broad array of uses as targeting agents, lectin inhibitors and drug delivery systems. Glycopolymers can be synthetic from bottom-up processes (such as Reversible Addition−Fragmentation chain-Transfer polymerization, RAFT; or Atom transfer radical polymerization, ATRP) or naturally occurring polymers that are used as is or modulated to enhance or modulate their properties (178–180). Glycopolymers have played major roles in the fields of general glycobiology and chemistry, as well as medicinal glycoscience. There are, obviously, many naturally occurring glycopolymers and a host of synthetic molecules that have been studied in a wide variety of contexts. Many natural polymeric materials have unique self-assembled properties and folds that categorize them as “nanoparticles”. A relevant example is the group of nanocellulose structures (nanocrystals, nanofibrils and bacterial nanocellulose) derived from different sources and where each have been employed in a wide range of applications, including those in biomedicine (181). Other polysaccharides such as starch, dextran, pullulan and chitin can all form nanostructures under appropriate conditions (182–184). The following brief discussion will outline select studies on these nanostructures and the reader is referred to the previous reviews (refs. 172-174) for details of glycopolymers in immunotherapy.

**Figure 6:**
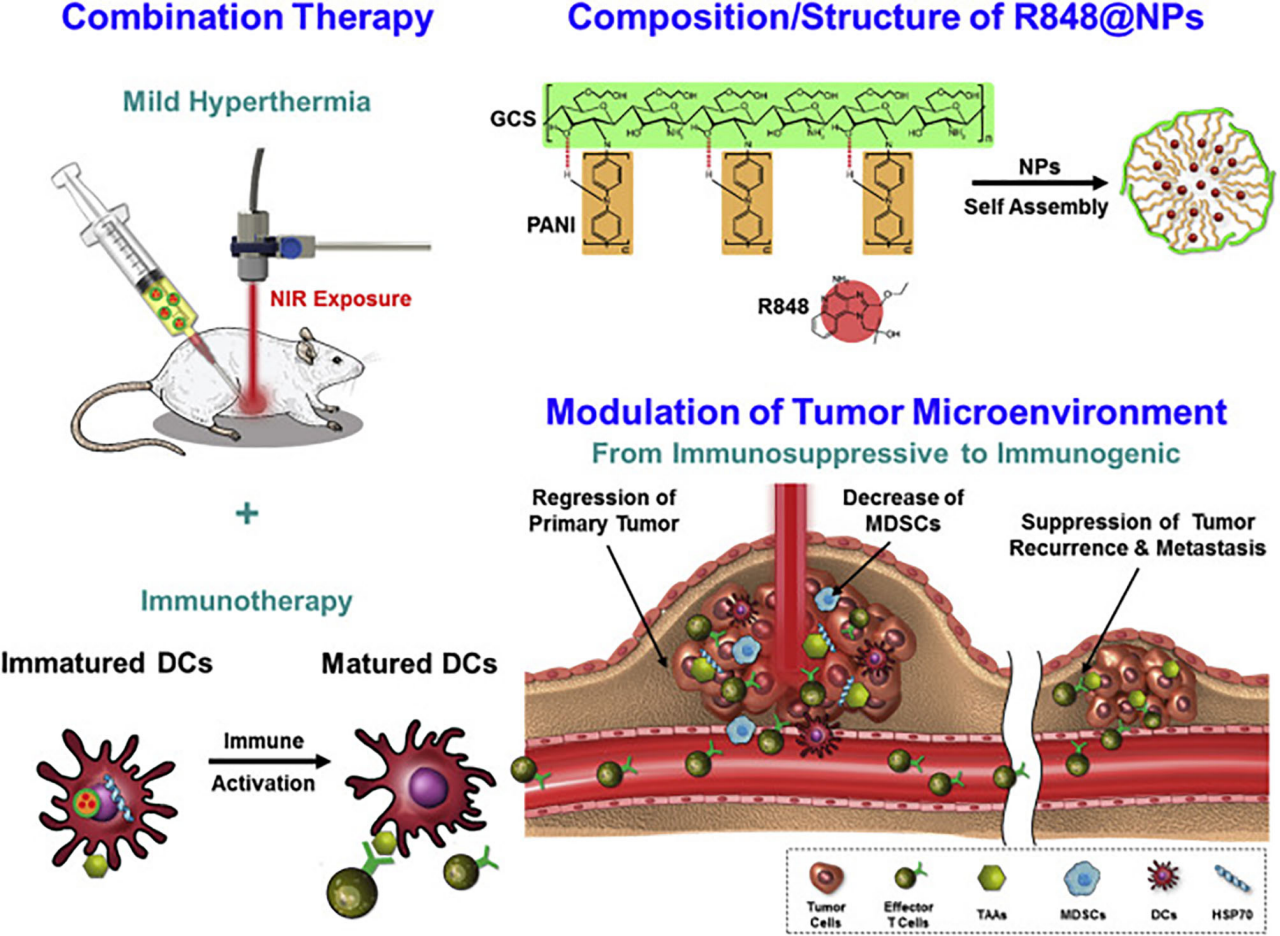
Design of photo-ablative and immunomodulating chitosan-based nanoparticles based on the activity of polyaniline and R848. Reprinted with permission from Elsevier (186).

Natural polysaccharides are ideal platforms for biomedical applications: They are biodegradable, biocompatible and mostly non-toxic. Designing delivery or biologically active systems from these structures can hence be more predictable and perhaps more easily approved for clinical use if sufficiently efficacious. Chitosan, prepared by the partial deacetylation of chitin (a polymer of β-1,4-linked N-acetylglucosamine), is perhaps most promising, as it is considered safe by the FDA its structure and properties can be modulated by controlled deacetylation. An interesting application was reported by Shi, et al., who designed a mannose-coupled chitosan nanoparticle in a whole tumor cell lysate-based vaccine preparation (185). The authors were able to prepare chitosan nanoparticles that were loaded with tumor cell lysates from B16 melanoma cells. To this was coupled a mannose-alginate conjugate for targeting the mannose receptor on immature dendritic cells. The “dual-sugar”-based vaccine preparation promoted antigen uptake by and maturation of bone-marrow-derived dendritic cells, enhanced the CD8+ response *in vivo* which led to a potent reduction of tumor burden in a mouse melanoma model. In another application, an ingenious nanoparticle derived from a glycol-modified chitosan particle was used for low temperature hyperthermal treatment of tumors and simultaneous immunomodulatory effects (186). A glycol-chitosan derivative was covalently linked to a polyaniline scaffold to create an amphiphilic polymer containing a conductive polymer (polyaniline) for efficient photothermal conversion when applied intratumorally. Addition of the TLR7/8 agonist, R848 (Resiquimod) allowed for self-assembly where a biocompatible polymer encased a small organic molecule within its hydrophobic core. When injected into murine CT26 colon carcinoma tumors, this material allowed for photoablation while also affecting immune stimulation *via* the TLR agonist. A potent immune memory was generated since re-challenge with CT26 tumors after initial therapy protected mice form tumor further growth. The design and outline of experiments are shown in **Figure 6**. These constructs were compared to “empty” nanoparticles (those without R848) and the authors showed a potent synergistic effect of using the “loaded” particle as opposed to the one simply bearing the thermo-conductive polymer. Multifaceted designs such as these have a strong potential for clinical use if starting with biocompatible and non-toxic polymers.

The nanoparticles that can be produced from starch, cellulose, alginates of chitosan can all have effects on the immune response when they are used to treat various immune cells. This is actually a fascinating feature of many natural polysaccharides: Size, shape and even minor forms of modification can dramatically affect the types of cytokine gene expression that they trigger in the presence of various types of monocytes or dendritic cells. For example, when Torres, et al., compared microfilms of potato starch (100’s of microns long) (187) to nanoparticles prepared from starch of the same potato family (40-60 nm) (188), a very different cytokine profile was generated by each treatment. Differential results are also obtained when using various forms of chitosan, carrageenan and alginate particles when after treatment of various immune cells (see Torres (184), and references therein for more information)

#### 4.1.5 Dendrimers

**Figure 7:**
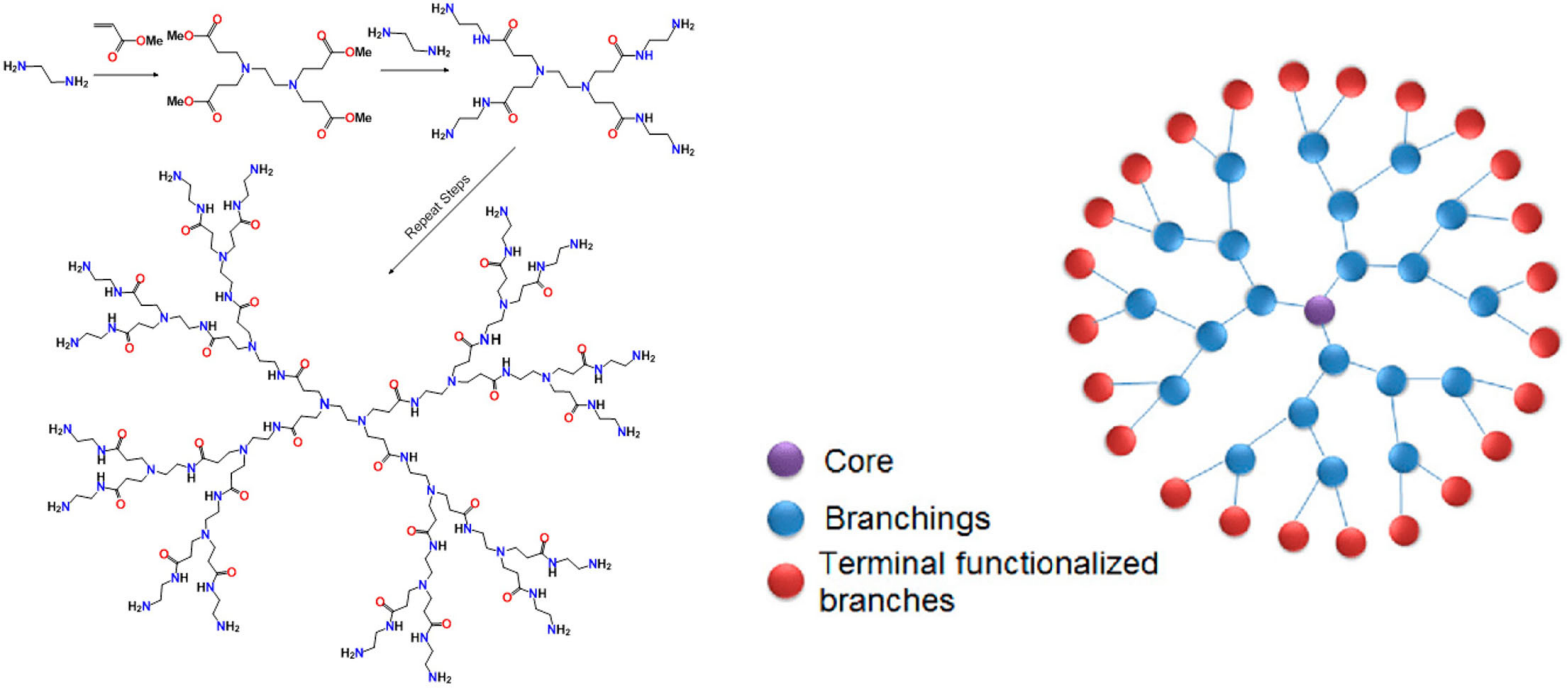
Basic synthetic scheme for construction of a PAMAM dendrimer (left). Schematic representation of a 24-mer dendritic structure.

Dendrimers are perhaps one of the more mature technologies for the preparation of multivalent glycan-based systems. Dendrimers are “treelike” (from the word Dendron) structures that can grow from only 2-4 functional groups to many, usually in multiples of 2 (189). Poly(amidoamine), or PAMAM dendrimers are the most common, comprised of amine and amide functionalities that begin with ethylene diamine reacting in a Michael fashion with methyl acrylate. This creates a tetra-carboxyl core that can double in size with subsequent additions of the diamine to form amine-terminated amides. Double addition to acrylate continues the process (**Figure 7**). Each addition is called a “generation” (0, 1, 2, 3….) and most of these are now commercially available. While the word dendrimer usually conjures up a “spherical” arrangement as shown in the right of **Figure 7**, it also can refer to any tree-like projection of multiple functional groups, such as one tetrameric branch of the 16-mer structure on the left of **Figure 7**.

As one can imagine, a dendrimer can be terminated with ether amines or a variety of other functional groups that may be used for 1) ligand conjugation chemistry, 2) display of charge to facilitate ionic interactions (such as adhesion of nuclei acids to polycationic amine-terminated dendrimers) or 3) encapsulation of metals (so-called metallo-dendrimers). Glycans are one such molecular family that have been attached to dendrimers for application such as lectin binding, cell-targeting and inhibition of cell adhesion. Arguably, the field of glycodendrimer technology was pioneered by the Roy group in Montreal beginning more than 25 years ago (190). His group have attached many tumor antigens and other carbohydrates to dendrimers for a variety of applications, including 1) defining density and molecular arrangement in lectin-sugar interactions (191, 192), 2) inhibiting these interactions in infectious disease (193) and 3) as antitumor vaccine platforms (25, 33, 194, 195). Dendrimers have now emerged as bona fide scaffolds for vaccines against many diseases, including cancer. As with many of the concepts described above, there is no shortage of recent reviews on this subject alone (25, 196–205). Thus, in the spirit of this review, the next paragraph will highlight some intriguing research of the last few years.

As mentioned above, dendrimers can come in different “flavors” that can nucleate different topologies depending on the number of branches and the specific chemistry used to create the structures. An early iteration was developed by Tam where he attached a lysine protected with a Boc group at both the α- and ε-nitrogen atoms to a β-alanine loaded resin (206). Deprotection and addition now of 2 similarly-protected lysine residues continued the growing cycle. He called these “multiple antigen peptide” (MAP) systems where an antigenic peptide would be synthesized on each of the nitrogen atoms of the dendritic structure. This system was utilized by Bay, et al., to attach both a Tn antigen TACA tripeptide and a CD4^+^ T-cell epitope as a vaccine against breast cancer (194). They showed that antisera from mice immunized with their construct could kill Jurkat cells by antibody-dependent cellular cytotoxicity (ADCC). Commendable on the author’s part is their conformational analysis of the peptides on the dendrimer by NMR. They found mostly a random coil distribution but some enhanced stiffening around the glycosylation sites. They attributed the enhanced activity of the multivalent system over the monomer to the possible clustering of the MGL receptor on APC’s caused by interaction with multiple copies of the TACA glycopeptide. A slight twist to this dendrimeric design is the formation of cyclopeptide-based scaffolds. A recent example of this was presented by Renaudet and co-workers who prepared a system containing both the Tn and TF TACAs on hexadecavalent cyclopeptide system (207). In a synthetic strategy that used a convergent approach to orthogonally protect different branches of the system, they were able to mix and match addition of the TF and Tn antigens for a heterogeneous display of the TACAs. Binding to a Tn-specific antibody showed a distinct preference for specific arrangements on the MAP system. The same group has prepared similar systems bearing the sialyl-Tn TACA (208) as well as Tn conjugated in a natural linkage to serine and one replaced with an oxime unit, where they showed that the oxime-linked Tn had superior immunotherapeutic properties *in vivo* (209).

**Figure 8:**
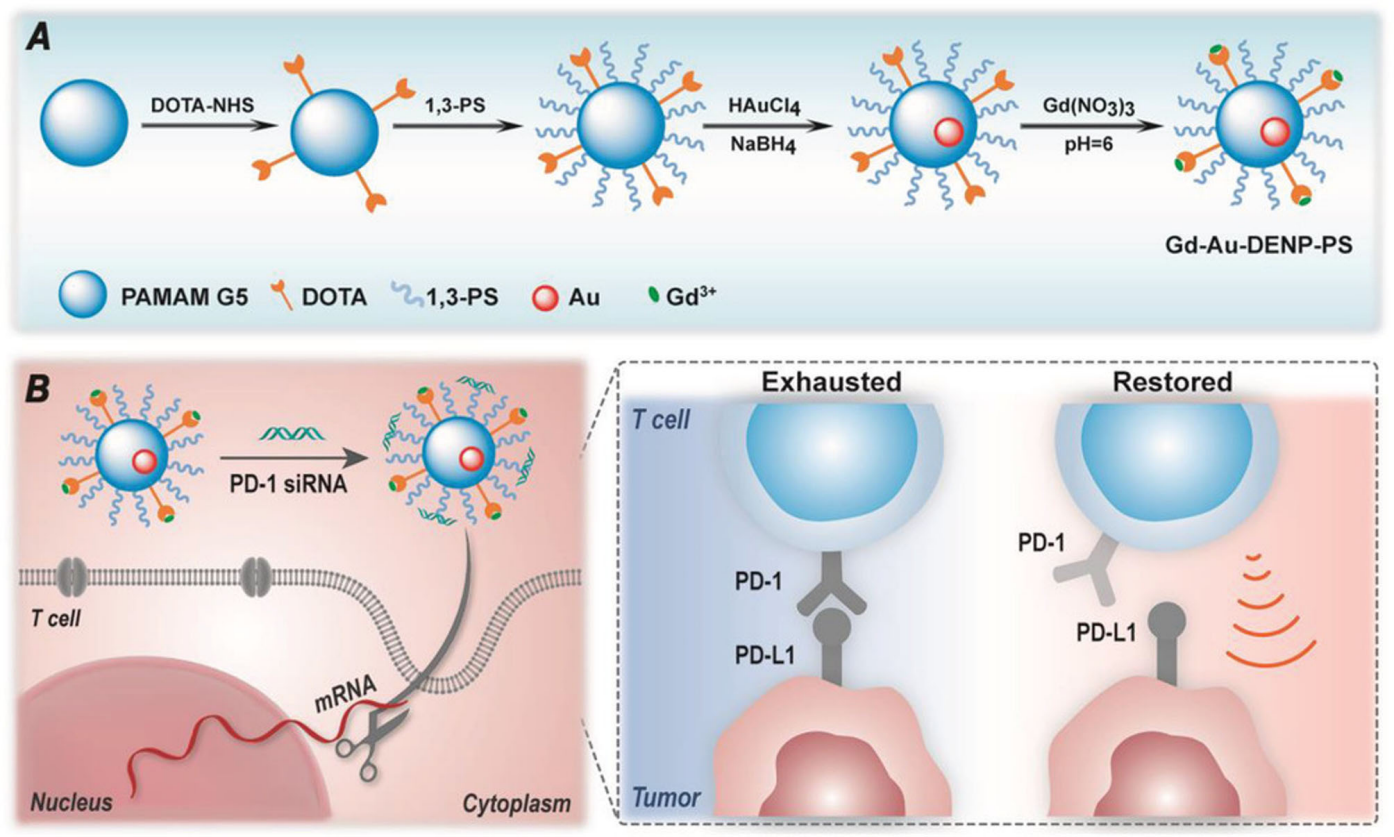
Schematic diagram of design of the dendrimer-encased AuNP from Shi et al. **(A)** Sequential coupling of DOTA and 1,3-propane sultone followed by AuNP formation and Gadolinium ligation. **(B)** Capping with PD-1 siRNA, followed by delivery to a T-cell results in reduced PD-1 expression and restoration of T-cell antitumor activity.

In a recent report, Sharma et al., showed that attachment of simple sugars (glucose, galactose and mannose) to hydroxyl-terminated dendrimers through a click chemistry approach, allows crossing of the blood-brain barrier into the Tumor Micro-Environment (TME) of glioblastomas to target overexpressed sugar transporters due to the Warburg effect to help internalize the dendrimer into Tumor-Associated Macrophages (TAMS) and microglial cells. This enhanced delivery to brain tumors should allow for delivery of small molecules drugs directly to difficult-to-access tumors. While not technically a “glycodendrimer”, an interesting report by Shi and coworkers has shown that a dendrimer entrapped AuNP can reduce T-cell exhaustion *in vivo* through the delivery of siRNA against the immune checkpoint molecule PD-1 (I consider nucleic acids part of the “glyco” family)! (210). This group has pioneered the use of these dual nanoparticle constructs since the AuNP core imparts altered conformational properties to the ligand presentation on the dendrimer (211). The ingenious design is based on the use of both 1,4,7,10-tetraazacyclododecane-N,N′,N″,N′″-tetraacetic acid (DOTA) and 1,3-propane sultone in subsequent additions to a dendrimer to create a ligand for gadolinium based MRI imaging and a zwitterionic cap where siRNA can be ionically trapped facilitating its delivery (**Figure 8**). In a more carbohydrate-based application, they have also use cyclodextrin-coated dendrimer-AuNPs to deliver siRNA to glioblastoma cells (212).

Finally, the use of dendrimers has been commercialized in the formation of companies to develop these platforms for clinical use. One company that is trying to market glycodendrimers is GlycoVax in Montreal. Based on technology of Professor Roy, Glycovax (https://glycovax.com/) is developing vaccines based on glycan-decorated dendrimers against COVID-19.

### 4.2 Reprogramming the Tumor Microenvironment (TME)

The immunosuppressive tumor microenvironment is a challenging obstacle to overcome when attempting to develop effective immunotherapies. A primary feature of the TME is the presence of immunosuppressive and tumor-promoting M2-polarized tumor-associated macrophages (TAMs). These are characterized by an anti-inflammatory cytokine profile that favors proliferation and tissue regeneration. Alternatively, the M1 phenotype is pro-inflammatory and antitumor, with high production of IL-12, nitic oxide, and reactive oxygen species (ROS). M2 TAMs release high amounts of IL-4 and IL-10, as well as upregulate C-type lectin receptors such as Dectin-1 and the Mannose receptor (MR) (213). Polarization between these two subtypes is dynamic and regulated by a series of cell-surface receptors, signaling pathways and pathogen-associated molecules such as LPS and other TLR agonists. Many signaling pathways such as those associated with IRK/STAT, GM-CSF, oxygen/ROS levels (hypoxia), various micro RNAs and carbohydrate receptors have been shown to be involved in the programing of either the M1 or M2 state, where the specific physiological milieu can swing this in one direction or the other. Better understanding of these processes and the ability to reprogram the M2 phenotype toward M1 has been an innovative strategy in recent tumor checkpoint-based immunotherapy (214).

Thus, it is perhaps not surprising that a variety of strategies have been employed to use nanomaterials, especially glycopolymer-based systems, where targeting of either TLR’s or CLR’s may direct polarization of one state or the other. Recent work by Weissleder’s group has shown that delivery of Resiquimod, R848 (*vide supra*) encased in β-cyclodextrin-based nanoparticles could repolarize M2 macrophages to M1 and enhance tumor suppressive mechanisms (215). Combination therapy with anti-PD-1 checkpoint antibodies was synergistic in shrinking MC38 mouse colon adenocarcinomas. Slightly outside the sphere of nanotechnology, microparticles coated with mannose and metformin, a diabetes drug that has been also shown to reprogram TAMs, were able to reprogram M2 macrophages and boost antitumor immunity (216). In a separate indication, what were considered “glycocalyx-mimicking” polymeric nanoparticles displaying different monosaccharide ligands for CLR’s were prepared in a block-copolymer fashion and self-assembled onto polystyrene beads. The nanosystems displaying fucose, mannose or galactose were all able to repolarize M2 macrophages to M1 both *in vitro* and *in vivo* (217). The same group showed similar results with anti-PD-L1 checkpoint inhibition synergy with polymers prepared by RAFT polymerization with galactose and mannose units. An application of microRNA delivery was studied where miR-125b was delivered specifically to a pancreatic ductal adenocarcinoma (PDAC) model using a self-assembled hyaluronic acid/PEG/polyethyleneamine nanosystem to affect repolarization in both *in vitro* and *in vivo* models of these PDACs (218). Finally, it was shown that a β-1,6 linked glucan from the fruiting body *Amillariella Mellea*, an edible fungus used in traditional Chinese Medicine can skew IL-4 induced M2 macrophages to M1 through a TLR2-mediated mechanism (219).

There are many other nanosystems that can also repolarize M1 to M2 that are not carbohydrate-based (220–229).

### 4.3 Ligand Chemistry, Number and Density

As mentioned above, many biological processes, and in particular ones that involve cellular glycans, involve binding of multiple copies of a “ligand” with a “receptor”. Multivalency is nature’s way of potentiating the effect of a specific interaction, such as an aspect of a specific signaling pathway or the adhesion of a cell to a protein or another cell. There is no universal method for determining coverage of any particular glyconanosystems since the chemistry used to prepare each entity, along with the morphology of shape and size of each system varies widely. There have been some standard carbohydrate analysis methods that have been used along with other more underdeveloped ways of approaching this question. Yan and coworkers have used the colorimetric anthrone/sulfuric acid method to determine carbohydrate content by a standard curve calibration method (230). We have used the phenol/surfuric acid system for determining polysaccharide coverage for our β-glucan AuNPs (172) Soledad Penades whose group pioneered the use of AuNPs in biomedical applications used quantitative ^1^H NMR to determine percentages of carbohydrate and linkers in hybrid-conjugated AuNPs (231). Yan has used quantitative ^19^F NMR to quantitate carbohydrate that were photochemically attached to perfluorinated aromatic residues on silica nanoparticles (232). Thermogravimetric analysis can also be used by determining elemental content of the organic matter attached to the particle (233). This is useful for nanoparticles that are homogeneously coated with glycans.

It is difficult to quantitate the proper arrangement and ligand placement that may be optimum for a specific biological response. As has been alluded to, multivalency is highly important in carbohydrate-macromolecule interactions, and many times, the adage “the more the merrier” holds true, especially when comparing multivalent displays with a monomeric sugar. However, a recent review sums this up nicely in the abstract of the article. After stating that many studies have been performed on ligand density, multivalent binding, cell internalization and size/shape considerations, the following sentence states: “*Although such experimental studies are very insightful, information is limited and confounded by numerous differences across experimental systems*” (234). Although this manuscript was not directed at glyconanoparticles, many of same features may hold true for sugar based systems. In fact, sugar-based constructs may even offer more complexity since high affinity/avidity carbohydrate-protein binding *requires* multivalency. There are however several examples of nanoparticle design where density and distance requirements are key to simple protein binding or stimulation of a biological response. The following section expands on this to morphology.

### 4.4 Size and Shape?

The previous paragraph begs the question of how the morphological properties of nanoparticles affect their biological activity, especially with respect to immune cell targeting, cell penetration, vaccine effectiveness and cytokine release profiles. There have been a handful of useful studies that make predictions as to what type of shape and/or size will be taken up more efficiently by various mammalian cells. Early work by Albanese and Chan showed that different size AuNPs of either spherical or rod-like shape with different aspect ratios can be taken up differentially by Hela cells. Their work was with “naked” citrate-stabilized AuNPs so the uptake was affected by non-specific protein binding of each particle. Later work by Odom showed that 50 nm AuNP spheres and 40 nm gold nanostars coated with siRNA were taken up more efficiently into endosomes of U87 glioblastoma cells than 13 nm AuNPs (235). Recent work by Xie, et al., compared gold stars, rods and triangles for uptake by the RAW264.7 macrophage cell line. They prepared uniform nanoparticles in all three shapes coated with methylpolyethylene glycol (mPEG) to stabilize the particles, reduce protein binding and maintain biocompatibility. All the nanoparticles were non-toxic to the cells up to 40 ug/ml concentrations. Triangles were the winner, and it was shown that each shape used a different endocytic pathway to enter the cells (236). In the same year, the group of Kikkeri in Pune prepared gold nano spheres, rods and stars and tested their toxicity and biodistribution in zebrafish (237). The nanoparticles were coated with simple a combination of short PEG units terminated by a fluorescein derivative and another molecule containing the same PEG chain but now terminated by an α-mannose unit, making this study more relevant to the glycol-nanotechnology field. All particles were also non-toxic to the animals and their biodistribution was examined by Inductively-Coupled Plasma Mass Spectrometry (ICP-MS). The data showed that rod shape particles were rapidly taken up by various organs but also cleared faster (48h), where star shaped particles remained in the organs for much longer periods of time. Increased uptake in the digestive tract was attributed to mannose receptors such as dendritic cell-specific intercellular adhesion molecule-3-grabbing non integrin (DC-SIGN) in this organ as well as the heart. The authors postulated that the slower clearance of the star shaped particle may make them more useful as a therapeutic delivery system. The same authors have recently posted a preprint of a similar study that is much more appropriate to the subject of this review. They prepared sphere, rod and star-shaped AuNPs coated with a trivalent Tn-bearing glycopeptide from MUC1 (238). They used the TLR9-agonist CpG-deoxyoligonucleotide as a co-surface molecule on the nanoparticles as an added adjuvant. They examined the effect of shape and adjuvant coating to 1) cellular uptake by murine dendritic cells, 2) Cytokine production in a DC/T-cell model and, 3) antibody production *in vivo*. Quite interestingly, they showed that uptake and immune response are “decoupled”, as rod-shaped particle were taken up by cells more efficiently but generated the weaker immune responses related to both cytokine stimulation and antibody titers. Although this paper has not yet been peer- reviewed, this would be the first study that offers a guide of to how more efficiently design gold nanoparticles vaccines with immunologically-relevant TACA glycopeptides, based on shape and adjuvant selection.

There are many other reports and nanoparticle systems that have been used to target glycan binding proteins or constructed of carbohydrates/polysaccharides that are too numerous to mention in this review. Provided here is a recompilation (not comprehensive)! of reviews only from 2021 that directs the reader to some of this work (24–27, 78, 82, 110, 239–246).

## 5 Conclusions and Future Outlook

There are few disagreements today of the importance of cellular glycans in a host of biological processes. Carbohydrate structures that are displayed in various different ways on a cell, all contribute to that cell’s proper and sometimes awry functions. Understanding the molecular mechanisms of how these structures interact with cells, proteins, lipids or other carbohydrates will allow researchers to develop probes, agonists and inhibitors of processes that are important to control proper cellular function that are propagated through glycan-based mechanisms. In tumors, the role of TACAs and other tumor-associated sugars has been elucidated for many systems, and researchers have made defined inroads into modulating these pathways to better treat neoplastic disease. This treatise also highlights nanotechnology in these roles, where, I believe, the development and eventual approval of glyco-nanosystems to reprogram and inhibit tumor signaling pathways is the future of glycan-based anticancer medicine. Three areas that have been discussed here and are high priority for future design are: 1) Vaccines. With the success at least up to early clinical trial, vaccines have the potential to revolutionize the cancer therapy landscape. One of the main issues, I believe, is antigen selection. The immunogen must be not only highly tumor selective, but truly needs to mimic the tumor-specific *presentation* of the molecules. That is, the use of techniques such as CryoEM and mass spec imaging may offer clue as to how some of these antigens are presented on a molecular level, and hence structural similarities can be closely mimicked through the use of chemical or chemoenzymatic synthesis. 2) Inhibitors of glycan-protein interactions. There are many relevant binding events that are glycan-mediated that contribute to tumor aggressiveness and better inhibitors that are well defined and selective can prolong life and prevent tumor metastasis. Nanoparticles can contribute greatly to the design of more potent and selective inhibitors that may be appropriately formulated for drug approval. Being a new area of therapy, regulatory agencies like the FDA are still formulating their guidelines for the proper requirements that a nanotechnology system must meet to progress to the stage of an “investigational new drug” (IND). Finalizing these guidelines will accelerate future approvals. The nanotoxicology program at the FDA is an important center for help with this endeavor. At the same time, researchers need to improve their formulations and toxicological evaluations prior to submission for an IND. 3) The development of glycan and glycopeptide-based CAR-T cell therapy. The success of CAR-T therapy to this point makes development of glycan-based CAR-T cells even more relevant, since I believe as was intimated in point #1, the antigens that the CAR-T cells target can be made more resemble tumors even more closely with proper glycan and peptide combinations. So far, there have been a handful of CAR-T cells directed at glycans or MUC1 glycopeptides, and their potential seems promising (46, 78, 243, 246, 247).

Ultimately, it seems clear that glycoconjugate/glycan/polysaccharide-based nanoparticles for therapeutic intervention in cancer immunotherapy, either alone or in combination with recently approved checkpoint inhibitors, has a bright future ahead.

## Author Contributions

JB devised the concept, did the research work and wrote the paper. The author confirms being the sole contributor of this work and has approved it for publication.

## Conflict of Interest

The author declares that the research was conducted in the absence of any commercial or financial relationships that could be construed as a potential conflict of interest.

## Publisher’s Note

All claims expressed in this article are solely those of the authors and do not necessarily represent those of their affiliated organizations, or those of the publisher, the editors and the reviewers. Any product that may be evaluated in this article, or claim that may be made by its manufacturer, is not guaranteed or endorsed by the publisher.

## Abbreviations

ADCC, Antibody-Dependent Cellular Cytotoxicity; AuNPs, Gold nanoparticles; BCG, Bacille Calmette-Guérin; CAR-T cells, Chimeric Antigen Receptor-T cells; CML, Chronic Myelogenous Leukemia; CRS, Cytokine Release Syndrome; CTL, C-type Lectin Receptor; DC-SIGN, Dendritic Cell-Specific Intercellular Adhesion Molecule-3-Grabbing Non-Integrin; GM-CSF, Granulocyte-Macrophage Colony Stimulating Factor; MAP, Multiple Antigen Peptide; MDSCs, Mononuclear-Derived Suppressor Cells; MGL, Macrophage Galactose-Type Lectin; PAMAM, Poly (amidoamine); SPIONs, Superparamagnetic Iron Oxide NanoParticles; TACAs, Tumor-Associated Carbohydrate Antigens; TAMs, Tumor-Associated Macrophages; TCGA, The Cancer Genome Atlas; TDM, Trehalose 6,6′-DiMycolate; TLR, Toll-Like Receptor.
